# T-large Granular Lymphocytic Leukemia: A Rare Diagnosis in a Young Woman With Fever, Necrotic Skin Lesions and Cytopenias

**DOI:** 10.7759/cureus.53468

**Published:** 2024-02-02

**Authors:** Mário Ferreira, Joana Paulo, Paulo Ramos, Carolina Padrão, Zélia Neves

**Affiliations:** 1 Serviço de Medicina Interna, Hospital Professor Doutor Fernando Fonseca, Lisboa, PRT; 2 Serviço de Anatomia Patológica, Hospital Professor Doutor Fernando Fonseca, Lisboa, PRT

**Keywords:** ecthyma, vasculitis, leukemia, lymphoproliferative disease, large granular lymphocytes

## Abstract

T-large granular lymphocytic leukemia (T-LGLL) is a rare lymphoproliferative disorder. The diagnosis is established by identifying an abnormally high number of clonal granular T lymphocytes in the peripheral blood and eventually in the bone marrow, in cases with medullary infiltration. The majority of patients present with symptoms related to neutropenia and this condition may be associated with autoimmune diseases in up to a third of cases. The authors describe the case of a 26-year-old patient admitted with subacute high fever and bullous dermatitis with necrotic lesions with central bullae. Analytically, she presented anemia and leukopenia with severe neutropenia of 200 cells/L. Skin lesions were compatible with ecthyma and the skin biopsy revealed aspects compatible with leukocytoclastic vasculitis. The myelogram and bone biopsy revealed hypoplasia of the myeloid line and a pathological T population of CD8+, TIA-1+ and granzyme B+, which were associated with compatible flow cytometry (CD3+, T-cell receptor (TCR) Alpha-Beta+, CD5+, CD2+, with loss of CD7 antigen expression) established the diagnosis of T-LGLL. The patient had a favorable evolution, with cytopenias almost returning to normal after two months. She began follow-up at a Hematology Reference Center, remaining asymptomatic without specific treatment considering the indolent course of the disease.

## Introduction

T-large granular lymphocyte leukemia (T-LGLL) is a lymphoproliferative disorder of mature CD3+ T lymphocytes, and must be distinguished from natural killer (NK)-large granular lymphocytic leukemia, a distinct entity [1]. When observed in peripheral blood smears, large granular lymphocytes are characterized as large cells with variably abundant cytoplasm and azurophilic granules [2]. Bone marrow cellularity varies but is slightly hypercellular in approximately half of cases, showing a mixed interstitial and intrasinusoidal infiltrates of small lymphoid cells without significant atypia. Interstitial reactive lymphoid aggregates predominantly comprised of T cells and B cells are frequent [1-3].

Most T-LGLL are of CD8 positive alpha-beta T-cells (CD8 T-LGLL) and have a pattern of post-thymic maturation, with a mature effector memory phenotype (CD3+/CD8+/CD57+/CD45RA+/CD62L-) [1,4,5]. Immunohistochemistry reveals the expression of CD2, CD3, CD8, T-cell receptor (TCR) beta, CD7, and cytotoxic granule proteins TIA-1, perforin, granzyme B and granzyme M. CD56 expression is infrequent, and CD5 is often absent. Expression of the NK-cell antigens CD16 and CD57 is frequent (80% and 90%, respectively) however, this feature is not disease specific [1,4].

Diagnosis of large granular lymphocyte (LGL) leukemia is given by the demonstration of clonality, which in T-LGLL can be provided by TCR gene rearrangement analysis, distinguishing reactive LGL proliferation from real leukemic proliferation [6]. Clonality may also be assessed by flow cytometry of TCR beta chain constant region (TRBC1) expression, being the majority alfa-beta variants, while 10% are gamma-delta variants [7]. The NK-cell associated receptors killer-cell immunoglobulin-like receptor (KIR) and CD94/NKG2 are expressed in over one-third of T-LGLL, indicating that these cells represent late stage fully differentiated cytotoxic T-lymphocytes [1]. NK-LGLL and T-LGLL might be indistinguishable by immunohistochemical analysis alone, needing clonality or molecular studies [1,6].

T-LGLL is an extremely rare entity, with an estimated incidence of 0.2-0.7 cases per million inhabitants per year, in Europe and the United States, respectively [6]. The diagnosis is generally made in the transition between adulthood and old age, with a median age at diagnosis of 66.5 years, with only 15% of patients diagnosed under the age of 50, being even rarer under 25 years of age. Several studies report similar incidence between sexes [4,8,9].

Although its etiology is not completely clear, it is thought that it may be associated with chronic antigenic stimulation in the context of other pathologies, given the mature phenotype of activated lymphocytes. T-LGL is associated with autoimmune diseases in one-third of cases, especially rheumatoid arthritis, and less frequently with neoplasms. It is assumed that prolonged stimulation in this context may cause LGL clonal expansion, associated with the deregulation of several pro- and anti-apoptotic signaling pathways. Recent studies point to the constitutional activation of STAT3, and more rarely of STAT 5b, as a key concept in the etiopathogenesis of LGL [6,10,11].

T-LGL is associated with the detection of asymptomatic cytopenias related to impaired hematopoiesis in about a third of patients, namely neutropenia (60-95%) and anemia (48%), with 80% of cases presenting moderate lymphocytosis (2 to 10x109/L). Thrombocytopenia (20%) appears to be more associated with the T-LGL form than NK-LGL [2,4,6,8,9].

The majority of patients with LGL present with symptoms related to neutropenia, namely fever and recurrent bacterial infections in 20 to 40% of cases [2,4,8]. Infections typically involve the skin, oropharynx, and perirectal areas. Splenomegaly may be present in up to half of cases, with hepatomegaly or lymphadenopathy being rarely reported [2,7-9].

Not all patients with T-LGLL require treatment at the time of diagnosis. A watch-and-wait approach is advocated in asymptomatic patients, without cytopenia or with mild changes, considering the indolent course of the disease [6,8,10]. Recurrent infections associated with neutropenia or anemia requiring transfusion, seen in up to a fifth of patients, are the most frequent manifestations that motivate the need for treatment. Especially in cases of associated autoimmune disease, specific treatment options are advocated with prednisolone, methotrexate, cyclophosphamide, cyclosporine or rituximab [2,4,5,7,10,12].

T-LGLL is seen as a chronic disease, with many patients remaining asymptomatic for several years. An average survival of 89% at five years is described, with the main cause of mortality being associated with serious infections [8,12]. Nevertheless, the prognosis of this disease is poorly established, particularly in young patients.

## Case presentation

**Figure 1:**
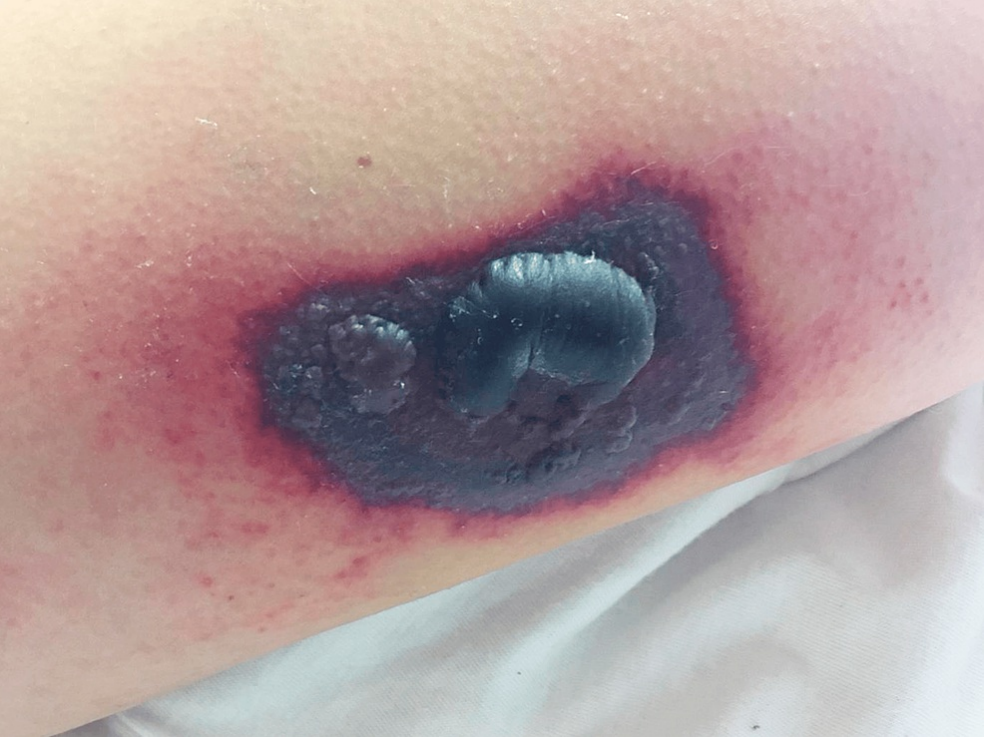
Necrotic skin lesion with relatively well-defined edges, central bulla and inflammatory halo on the right arm.

**Figure 2:**
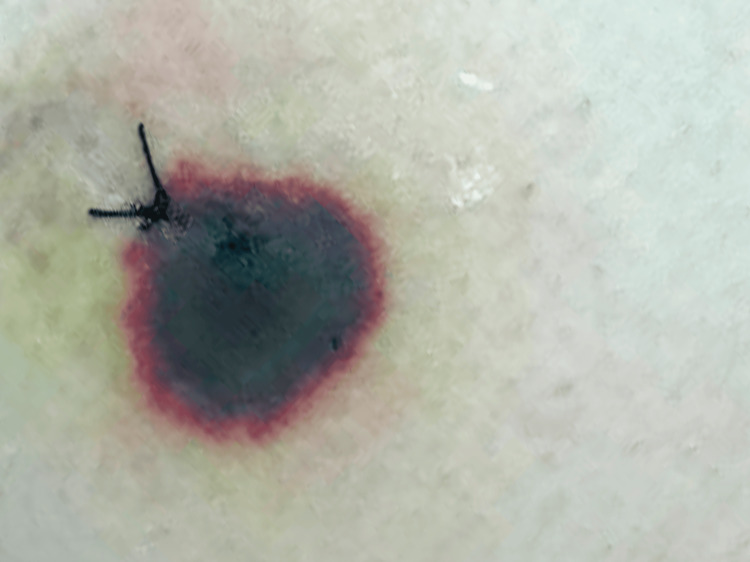
Early state of necrotic lesion on the right thigh, biopsied at its active edge.

**Figure 3:**
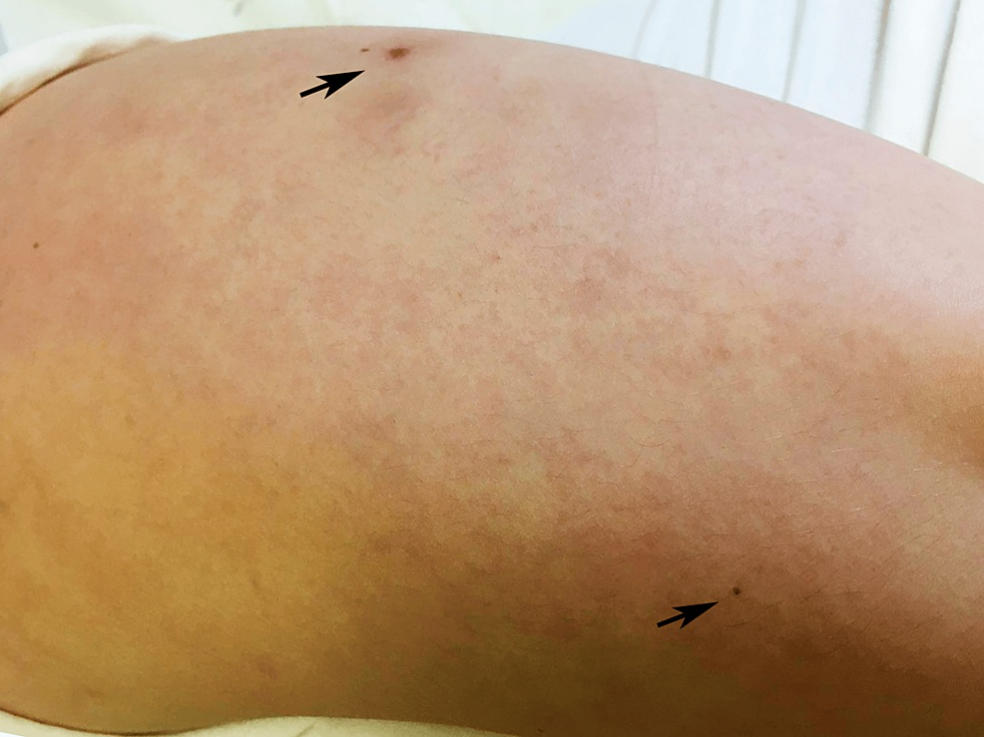
Livedo reticularis (left arm) preceding the appearance of painful necrotic plaques (black arrows)

We present the case of a 26-year-old caucasian woman, admitted due to subacute complaints of high fever (38.7-39.5ºC), otalgia and livedo reticularis in the lower limbs that evolved into painful necrotic plaques with central bullae that progressed to ulcers. She denied nocturnal diaphoresis, weight loss, headaches, neurological symptoms, myoarthralgias, jaundice, hematuria or recent viral infection. The patient had a history of migraines with frequent use of nonsteroidal anti-inflammatory drugs (NSAIDs). The epidemiological history was negative, namely rural contact and exposure to vectors and the family history was negative for malignant blood diseases. On physical examination, she presented fever, normotension, mucocutaneous pallor and no adenopathies. Three necrotic lesions were observed in the posterior region of the left leg, right buttock and left arm (Figures 1-3). No signs of haemorrhagic dyscrasia were found. The abdomen was painless, without ascitis or hepatosplenomegaly.

Initial lab results revealed hemoglobin (Hb) 10.8 g/dl, mean corpuscular volume (MCV) 77 fL, mean corpuscular hemoglobin concentration (MCHC) 32.3 g/dl, leukocytes of 3500x106, lymphocytosis 52%, neutrophils of 1500 cells/L and normal platelet count. The peripheral blood smear showed erythrocyte anisocytosis and lymphocytes with hyperbasophilic cytoplasm. Liver and kidney function tests and protein electrophoresis were within the reference values, lactate dehydrogenase (LDH) 141 U/L, ferritin 119 ng/ml, sedimentation rate 27mm, folic acid 1.56 ng/ml, vitamin B12 379 pg/ml, calcium 9.2 mg/dl, uric acid 3.1 mg/dl, total bilirubin 0.42 mg/dl and C-reactive protein 8.0 mg/dl. The urinary sediment was negative and the chest X-ray and ECG were unremarkable.

Additional studies include negative blood and urine cultures. Likewise, the main viral serologies (HIV, hepatitis C virus (HCV), hepatitis B virus (HBV), Epstein-Barr virus (EBV), cytomegalovirus (CMV) and parvovirus), venereal disease research laboratory (VDRL) and serology for Lyme disease were negative. The autoimmunity study, namely rheumatoid factor (<10 IU/ml), cryoglobulins and antiphospholipid antibodies, was negative. ENT and Ophthalmology examinations, as well as transthoracic echocardiogram, revealed no changes. The skin lesion on the left leg was drained, with the presence of neutrophilic exudate and a negative culture test. Two biopsies of skin lesions were performed, both compatible with leukocytoclastic vasculitis of small vessels with fibrin thrombi. Body CT showed mild hepatomegaly, without adenopathies, splenomegaly, pleural effusion or ascites.

**Figure 4:**
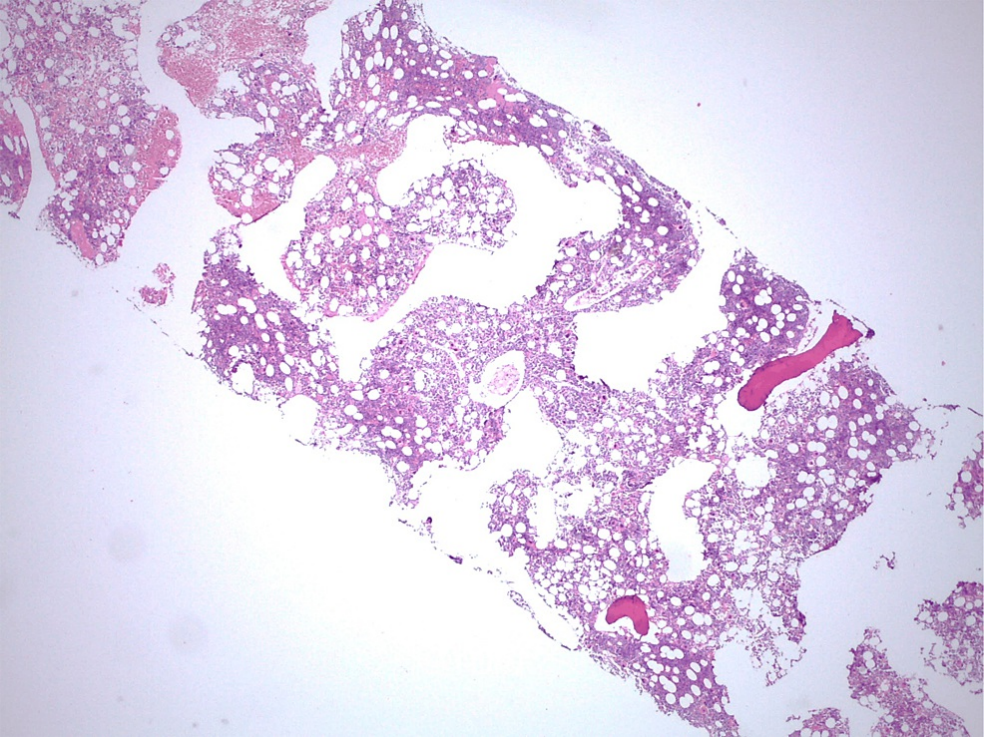
Bone marrow slightly hypercellular, H&E (250x).

**Figure 5:**
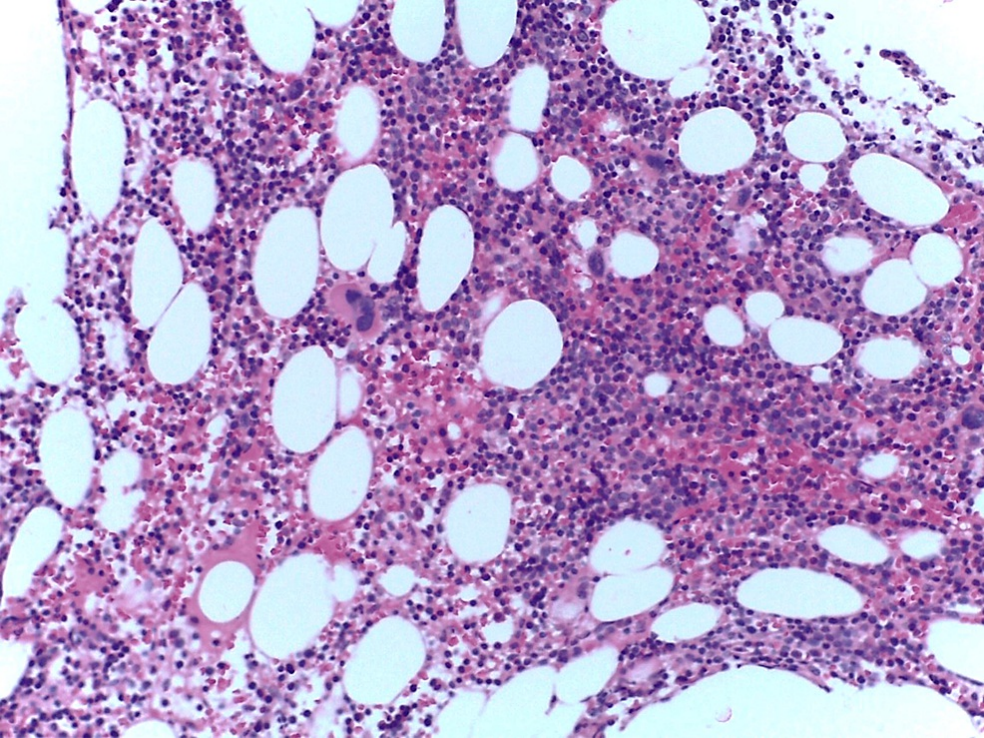
The bone marrow has an increase in lymphoid cells, the neoplastic cells are indifferentiable from the reactive cells.

**Figure 6:**
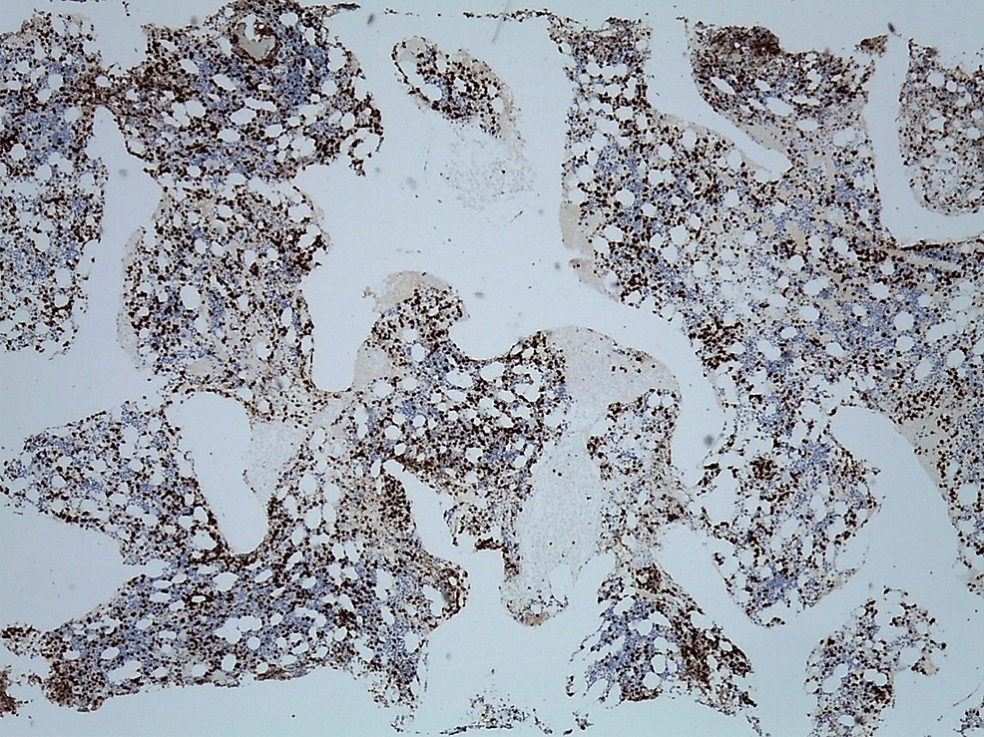
Immunohistochemical study with CD8 shows an abnormal bone marrow infiltration.

**Figure 7:**
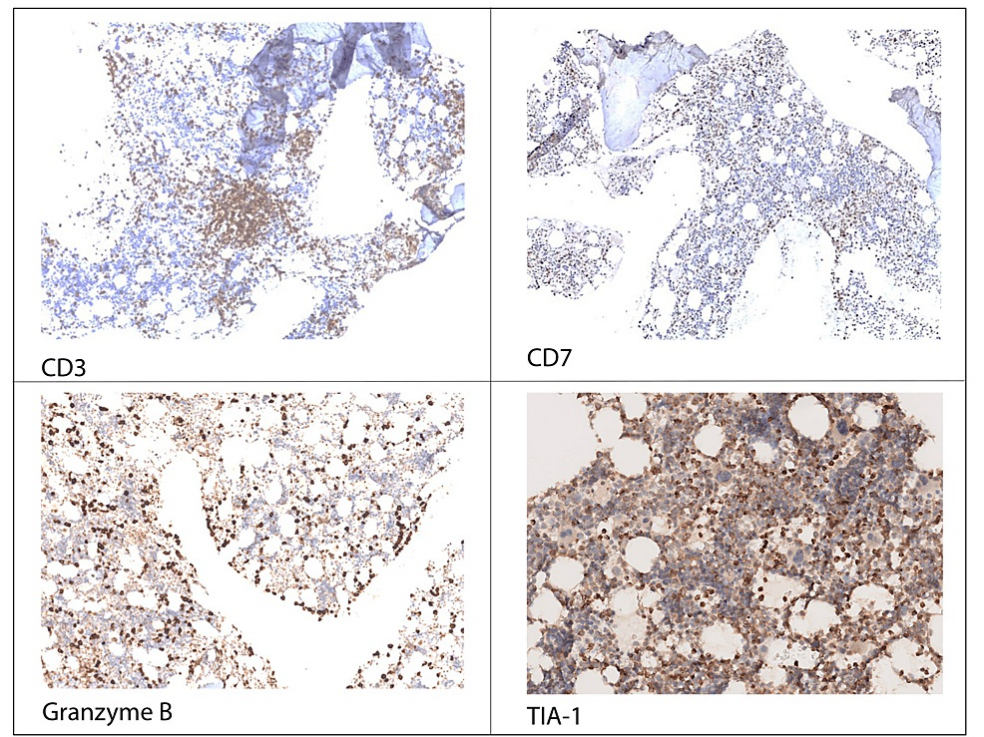
Immunohistochemical analysis of CD3, CD7, Granzyme B, TIA-1, CD3 is present in neoplastic and reactive cells, CD7 is decreased in the neoplastic cells, Granzyme B and TIA-1 are showing the neoplastic cells.

Considering the persistence of cytopenias, a myelogram was performed, which revealed marked hypoplasia of the myeloid line. The bone marrow biopsy (Figure 4) identified an abnormal infiltrate of lymphoid cells (Figure 5), predominantly pathological T (CD3+, CD2+, CD5+) cells, sometimes intra-sinusoidal, with a subpopulation of cytotoxic phenotype (CD8+/TIA-1+/Granzyme-B+) (Figures 6, 7) with aberrant expression of CD57, loss of CD7 expression and absence of CD56 and EBV-encoded RNA (EBER) expression.

Flow cytometry identified a subpopulation of T lymphocytes (CD3+/TCR alpha-beta+/CD5+/CD2+) with loss of CD7 antigen expression. These aspects were suggestive of medullary infiltration by T-LGLL.

The patient was referred to a Hematology reference center, where she remains under follow-up, and has not yet started treatment. There was residual healing of the skin lesions and analytical normalization two months after discharge (Table 1).

**Table 1:** Serial evolution of laboratory results throughout hospitalization. The nadir of neutropenia on the 10th day of hospitalization correlated with the peak of ecthyma lesions.

Lab	Day 1	Day 7	Day 10	Day 27	Day 34	Reference values
Hemoglobin	10.8	10.4	9.7	10.4	11.4	11.5-16.5 g/dL
Leukocytes (x10^9^)	3.50	3.00	3.00	4.20	4.91	4.0-11.0 x10^9^
Neutrophils (% of total leukocytes)	1500 (42.8%)	400 (12.4%)	200 (6.4%)	1400 (34.5%)	1850 (37.7%)	1800-6900 cells/L
Lymphocytes (% of total leukocytes)	1700 (48.6%)	2200 (72.8%)	2000 (67.8%)	2100 (50.6%)	2490 (50.7%)	1200-3300 cells/L
Platelets (x10^9^)	217	386	383	376	326	150-400 x10^9^

## Discussion

Considering the clinical context, the differential diagnoses based on the constellation of fever, necrotic skin lesions and marked cytopenia of relatively acute onset in a previously healthy patient included infection, vasculitis and hematological disease.

The possibility of tick-borne disease could be evoked by the necrotic lesions that mimicked inoculation eschar. Even so, its multiple nature, the absence of a skin rash and a compatible epidemiological history excluded this hypothesis, with negative serology for Mediterranean spotted fever. Likewise, the epidemiological history did not identify any spider bites, which can be manifested by similar dermonecrotic lesions.

The exuberant and unusual skin lesions were clinically compatible with ecthyma due to their main characteristics: several lesions, predominantly on the lower limbs, began with a vesicular pustule that increased in size with a hemorrhagic crust and evolved into an ulcer with a necrotic base and healed slowly with a depressed scar. Cultural examination of the drained lesion did not isolate an infectious agent but was compatible with an acute inflammatory process. Skin infection was closely related to the nadir and prolonged duration of neutropenia.

The prominence of cytopenias (anemia and leukopenia) associated with fever and bacterial skin infection, probably secondary to marked neutropenia, increased the suspicion of malignant hemopathy, particularly uncommon forms of leukemia. Therefore, a differential diagnosis was made with non-Hodgkin's lymphoma or chronic lymphocytic leukemia, which can manifest with predominant splenomegaly and minimal lymphadenopathy, aspects that were otherwise absent in our patient.

The diagnosis of T-LGLL was evoked by immunohistochemical study on bone biopsy with demonstration of T cells expressing CD8+, TIA-1+ and Granzyme B+ and established by demonstration of T cell monoclonality by flow cytometry. At this point, the distinction between LGL-T leukemia and reactive proliferation of LGL was possible through the identification of medullary infiltration and expression of TCR alpha-beta, which does not occur in transient or reactive processes.

## Conclusions

In addition to its interest as an extraordinarily rare entity, this clinical case illustrates the importance of diagnostic suspicion of T-LGLL at an unusual age, in the presence of cytopenia, including neutropenia of unexplained cause, associated with fever and bacterial infection. The relationship between T-LGLL and other diseases, namely autoimmune in one-third of cases, reveals the importance of its active investigation. In the case presented, the clinical history, physical examination and negative autoantibody panel did not suggest this association. Considering the indolent and chronic evolution of T-LGLL, a watch-and-wait approach is acceptable in asymptomatic patients, without cytopenia or with only mild changes, reserving immunosuppressive treatment for severe or refractory forms of the disease.
